# Antifungal Activity of Sodium New Houttuyfonate Against *Aspergillus fumigatus in vitro* and *in vivo*

**DOI:** 10.3389/fmicb.2022.856272

**Published:** 2022-04-26

**Authors:** Qian Zhang, Fangyan Liu, Meng Zeng, Jinping Zhang, Yanfei Liu, Caiyan Xin, Yingyu Mao, Zhangyong Song

**Affiliations:** ^1^School of Basic Medical Science, Southwest Medical University, Luzhou, China; ^2^Department of Clinical Laboratory, The Affiliated Hospital of Qingdao University, Qingdao, China; ^3^Molecular Biotechnology Platform, Public Center of Experimental Technology, Southwest Medical University, Luzhou, China

**Keywords:** sodium new houttuyfonate, *Aspergillus fumigatus*, ergosterol synthesis, antifungal agent, invasive aspergillosis

## Abstract

*Aspergillus fumigatus* is an important pathogen causing invasive aspergillosis, which is associated with high morbidity and mortality in immunocompromised people. However, the treatment of *A. fumigatus* infection is a growing challenge, owing to the limited availability antifungal agents and the continual emergence of drug-resistant strains. Drug repurposing is a potential strategy to solve this current problem. Sodium new houttuyfonate (SNH), derived from houttuynin, extracted from *Houttuynia cordata*, has anti-bacterial and anti-*Candida albicans* effects. However, whether it has anti-*A. fumigatus* activity had not been reported. In this study, the antifungal properties of SNH against *A. fumigatus*, including the standard strain AF293, itraconazole resistant clinical strains, and voriconazole resistant clinical strains, were evaluated *in vitro* and *in vivo*. Moreover, the potential mechanism of SNH was characterized. SNH exhibited significant fungicidal activity toward various *A. fumigatus* strains. SNH also inhibited fungal growth, sporulation, conidial germination and pigment formation, and biofilm formation. Further investigations revealed that SNH interfered with the *A. fumigatus* cell steroid synthesis pathway, as indicated by transcriptomic and quantitative real-time polymerase chain reaction analyses, and inhibited ergosterol synthesis, as indicated by cell membrane stress assays and ergosterol quantification. Moreover, daily gastric gavage of SNH significantly decreased the fungal burden in mice with disseminated infection (kidney, liver, and lung) and local tissue damage. In addition, the application of SNH downregulated the production of IL-6 and IL-17A. Together, these findings provided the first confirmation that SNH may be a promising antifungal agent for the treatment of *A. fumigatus* infection.

## Introduction

*Aspergillus fumigatus* is an opportunistic pathogen causing life-threatening infection in immunocompromised individuals (Gülmez et al., 2021). These infections can lead to a variety of pulmonary fungal diseases including allergic bronchopulmonary aspergillosis, chronic pulmonary aspergillosis, and invasive pulmonary aspergillosis (IPA; Pasula and Chandrasekar, 2021). Among them, IPA is the most common invasive disease. The infectivity rate of IPA in hematopoietic stem cell transplantation is 43%, and that in solid-organ transplant recipients is 59% (Latgé and Chamilos, 2019). Therefore, the treatment of *A. fumigatus* infection is a clinical challenge (Benedict et al., 2019). The current therapy for *A. fumigatus* infection involves triazoles, including isavuconazole, itraconazole (ITR), posaconazole, and voriconazole (VRC; Supplementary Figure 1). Although VRC is the drug of choice for the treatment of IPA disease, the death rate in azole-resistant IPA patients varies from 50 to 100% (Meis et al., 2016). Moreover, the resistance of *A. fumigatus* strains to triazole is continually increasing (van der Linden et al., 2015). Therefore, new antifungal agents are urgently needed, particularly for triazole-resistant strains. Although many investigations are being conducted to develop new and effective compounds, the bioactive molecules derived from existing drugs may be promising treatments (Zhang et al., 2021).

Sodium houttuyfonate (SH) and sodium new houttuyfonate (SNH) are modified compounds derived from the plant *Houttuynia cordata* Thunb (Supplementary Figure 1; Shao et al., 2012), an herbal drug with antibacterial, antiviral, and antifungal effects that is clinically used in Asia. Many investigations have revealed that SH has various biological and pharmacological activities such as anti-inflammatory activity in the respiratory tract and antibacterial activity against Gram-positive bacteria (Da et al., 2019); and anti-*Candida albicans* activity (Huang et al., 2015; Shao et al., 2017; Da et al., 2019). In addition, a previous investigation has demonstrated that SH and SNH possessed highly similar structures and anti-infective biological activities (Shao et al., 2012). However, SNH has better clinical value and higher chemical stability than SH (Zhao et al., 2020). Investigations have also confirmed that SNH exhibits antibacterial effects against *Staphylococcus aureus* and methicillin-resistant *S. aureus* (Lu et al., 2013; Li et al., 2020), *Streptococcus pneumoniae* (Yang et al., 2016), and *C. albicans* (Wu et al., 2020). Although SNH acts as an anti-inflammatory medicine and has various anti-bacterial and anti-*C. albicans* activities, the anti-fungal effect against *A. fumigatus*, particularly various triazole-resistant strains, has not been reported.

In this study, the effects of SNH against *A. fumigatus* strain conidial germination and pigment formation, fungal growth, sporulation, and biofilm formation were investigated *in vitro*. Furthermore, with transcriptomic and quantitative real-time polymerase chain reaction (qRT-PCR) analyses, stress assays, and the quantification of ergosterol, the mechanism underlying the effects of SNH were investigated. Additionally, we constructed a murine model of invasive aspergillosis (IA) and the antifungal activity of SNH was demonstrated.

## Materials and Methods

### Strains, Media, and Chemicals

The *A. fumigatus* standard strain AF293, ITR-resistant clinical isolates (AF1 and AF2), and VRC-resistant clinical strains (AF4 and AF5) were routinely grown on potato dextrose agar (PDA). Before each experiment, the strains used in this study, were cultured on PDA medium for 4 days at 37°C. The drugs, including ITR, VRC, and AmB, were purchased from *Macklin Chemicals* (Shanghai, China). SNH was obtained from *Fengyao Tonghui Chemicals* (Wuhan, China). ITR, VRC, and AmB were dissolved in DMSO to prepare a 5.12 mg/mL stock solution. SNH was dissolved in sterile distilled water with 0.05% Tween 80 to prepare a 5 mg/mL stock solution. All drug stock solutions were stored at –20°C.

### Minimal Inhibitory Concentration and Interactions Between Sodium New Houttuyfonate and Antifungal Agents

The minimal inhibitory concentration (MIC) of ITR, VRC, AmB, and SNH inhibiting growth of *A. fumigatus* were tested according to the reference Clinical and Laboratory Standards Institute M38-A2 document (CLSI, 2008; Espinel-Ingroff et al., 2010). The drug concentrations ranged from 0.06 to 32 μg/mL for both ITR and VRC, from 0.03 to 16 μg/mL for AmB, and from 0.0625 to 2 mg/mL for SNH. The conidia were dispersed in RPMI-1640 medium at a concentration of 0.4 × 10^4^ to 5 × 10^4^ cells/mL. The suspensions were then dispensed into triplicate wells of a 96-well microtiter plate. After incubation at 37°C for 48 h, MIC endpoints were defined as the lowest drug concentrations that caused complete visible inhibition of growth, as compared with the drug-free growth control. The criteria for determining the antifungal susceptibility of molds followed the CLSI M38-A2 document (Espinel-Ingroff et al., 2010). Experiments in each strain were performed in triplicate.

Interactions between SNH and antifungal agents (ITR or VRC) against the test strain were assessed with the MIC method as described above. The final drug concentration SNH ranged from 0.01 to 0.13 mg/mL, and those of antifungal agents (ITR or VRC) ranged from 0.03 to 32 μg/mL. The fractional inhibitory concentration index (FICI) was defined according to the M38-A2 document (Odds, 2003).

### Spot Dilution Assays

The sporulation of *A. fumigatus* was evaluated by assessment of the initial growth of a droplet of conidia from a serial dilution at final concentrations of 0 × (negative control), 1 ×, 2 ×, and 4 × MIC at two temperatures (37 and 28°C). Spot dilution experiments were performed with 3 μL of a 10-fold dilution series starting at a concentration of 10^5^ cells/mL spotted on PDA solid medium at the two temperatures for 96 h (37°C) and 120 h (28°C). Samples were collected from approximately the same three locations of colonies with an Oxford cup (Beijing Jitai Yuancheng Technology Co., Ltd., Beijing, China) and counted with a hemacytometer to estimate the average sporulation per square centimeter. Meanwhile, the conidial melanin/pigment, isolated from 4-day-old cultures with 2% NaOH, was purified and measured as described previously (Song et al., 2018).

### Effects on Germination

Fresh conidia were prepared with sterile phosphate buffered saline (PBS) and cultured at 37°C in liquid PDA medium at a concentration of 10^6^ cells/mL with various concentrations of SNH as described above. Then conidia were collected after 12, 16, and 20 h incubation. Germination was defined by a germ tube longer than the conidia diameter (Chen et al., 2020). The number of germinated conidia under a microscope (400 ×).

### Effect on the Metabolic Activity of Biofilm

The metabolic activity of biofilms was monitored with tetrazolium salt 2,3-bis (2-methoxy-4-nitro-5-sulfophenyl)-5-(phenylamino)-carbonyl-2*H*-tetrazoliumhydroxide (XTT; Macklin, Shanghai, China) reduction assay (Bugli et al., 2013; Bom et al., 2015; González-Ramírez et al., 2016). Briefly, for the biofilm formation assays, *A. fumigatus* spore suspension was prepared in RPMI-1640 medium supplement at a concentration of 10^6^ cells/mL with the above-described concentration of SNH, and the 96-well polystyrene plates were incubated at 37°C for 24 h. For the preformed biofilms, 200 μL of conidial suspension (10^6^ cells/mL) was added into each well of a microtiter plate and incubated at 37°C for 24 h. Next, the plate was washed with PBS three times. Then 200 μL SNH, at the above-described concentrations, prepared in RPMI-1640 medium was added. After incubation at 37°C for 24 h, the plates were incubated with XTT-menadione solution in the dark at 37°C for 2 h. Finally, the absorbance of the supernatant was measured at 492 nm (Pumeesat et al., 2017).

The confocal laser scanning microscopy (CLSM) was performed to observe biofilm developed on the coverslips (Shanghai Baiyan Bio-Technology Co., Ltd, Shanghai, China) in 12-well plates (Shanghai Huipan Industerial Co., Ltd, Shanghai, China). Method was following the previous investigation with slight modifications (Iwahashi et al., 2020). Briefly, the biofilms were washed three times with PBS and stained with calcofluor white stain (Sigma-Aldrich Trading Co., Ltd, Shanghai, China) supplemented with 20 μL 10% KOH at room temperature for 5 min. Fluorescent images were taken and analyzed by CLSM using OLYMPUS cellSens Dimension 3.2 (Olympus Co., Ltd, Tokyo, Japan).

### Time-Kill Assays

To investigate the effects of concentration and exposure time on the activity of SNH, we performed time-kill experiments and the methodology referred to previous studies with slight modifications (Bugli et al., 2013). All strains were grown in RPMI-1640 medium with a starting inoculum of 10^6^ cells/mL. The SNH working concentrations were 1 ×, 2 ×, 4 ×, and 8 × MIC. Samples were incubated at 37°C without agitation. At predetermined time points (0, 4, 8, 12, 16, 20, and 24 h) after incubation, the samples were transferred to 96-well plates, and their metabolic activity was determined with XTT reduction assays. The method to detect the metabolic activity was as described above.

### Propidium Iodide Staining

After treatment with SNH, propidium iodide (PI) staining assays were used to investigate the *A. fumigatus* cell membrane integrity (Chen et al., 2020). The inoculum of the AF293 strain was treated with the above-described concentrations of SNH and incubated at 37°C for 12 h. Then the samples were washed with PBS three times. PI (Solarbio, Beijing, China) was incubated with *A. fumigatus* at a concentration of 20 μg/mL for 30 min in the dark. The specimens were observed under a fluorescence microscope (Leica, Germany). Results represent three independent experiments.

### Transcriptomic and qRT-PCR Analysis

Conidia of the AF293 strain were cultured in RPMI-1640 medium with or without SNH (2 × MIC) for 12 h at 37°C. Then samples were collected, treated with liquid nitrogen, and stored at –80°C for collection of total RNA. Total RNA was prepared with RNAiso plus reagent (TaKaRa, Dalian, China) and reverse-transcribed into cDNA with a PrimeScript™ RT reagent kit with gDNA Eraser (TaKaRa). Illumina RNA sequencing was then conducted by Biomarker Technologies (Qingdao, China). To determine time-specific expression patterns of genes in the ergosterol synthesis pathway, we treated the AF293 conidia prepared in RPMI-1640 medium with 2 × MIC SNH and then incubated them at 37°C for 0, 6, and 12 h for RNA extraction. RT-qPCR was performed with a PrimeScript RT reagent kit with gDNA Eraser (TaKaRa, Dalian, China). Transcripts of the β-tubulin gene were used as an internal control. Transcript ratios were evaluated with the 2^–Δ^ ^Δ^ *^CT^* method (Vandesompele et al., 2002). The primer sequences are listed in Supplementary Table 1.

### Cell Membrane Stress Assays

Conidia of *A. fumigatus* AF293 were prepared in RPMI-1640 medium at a concentration of 10^6^ cells/mL with final concentrations of 0, 1/2 ×, and 1 × MIC SNH. Samples were incubated at 37°C for 12 h. Then a 3 μL aliquot containing 10^5^ cells/mL was inoculated onto PDA medium containing 0, 1, 2, or 4 μg/mL AmB. After inoculation at 37°C for 4 days, colony morphology was investigated with a digital camera (60-mm Macro lens, Canon Inc., Japan).

### Quantification of Ergosterol

To quantify the concentration of ergosterol, we incubated AF293 conidia in RPMI-1640 medium with different concentrations of SNH (1 ×, 2 ×, and 4 × MIC) at 37°C for 3 days. The mycelia were harvested and washed with PBS twice, as described previously (Pinto et al., 2011). Then 0.500 g samples were dispersed in 100% methanol for ultrasound-assisted extraction. The content of ergosterol was determined by high-performance liquid chromatography (HPLC; 1260 infinity II, Agilent). Ergosterol standard (Macklin, Shanghai, China) was dissolved with 100% methanol to a concentration of 100 μg/mL and used as a reference solution. The concentration of ergosterol was calculated with the following equations: (peak area of experimental group × concentration of ergosterol standard group)/peak area of ergosterol standard product. Experiments for each strain were performed in triplicate.

### Antifungal Activity *in vivo*

Male 6–8-week-old Balb/c mice (Chongqing Tengxin Biotechnology Co., Ltd., Chongqing, China) with a weight of 20–25 g was given food and water *ad libitum*. Mice were immunosuppressed by injection of cyclophosphamide at 200 mg/kg intravenously for 3 days (Denning et al., 1995, 1997). Then the mice were infected by injection of a 100 μL inoculum of *A. fumigatus* AF293 into the tail vein at a final concentration of 10^7^ cells/mL. After the successful establishment of an IA mouse model, treatment was started 2 h after inoculation and was continued for 7 days by gastric gavage with SNH at 10 mg/kg/d and 30 mg/kg/d. The same volume of normal saline and ITR with a concentration of 75 mg/kg/d were used in the control group.

Fungal burden and histopathology were used to evaluate the efficacy of SNH against IA. The liver, kidney, lung, and serum were collected after the mice were treated for 1, 4, and 7 days. One half of the collected liver, kidney, and lung samples was used to prepared dilutions of the homogenates and plated on PDA. The number of colony formation units per gram of tissue was determined after at least 48 h of incubation at 37°C. The other tissues were fixed in 10% methanol and embedded in paraffin. Thin sections were cut and stained with periodic acid-Schiff stain (PAS) for microscopic observations.

Cytokines were detected with a BD Cytometric Bead Array Mouse Inflammation Kit (Univ, Shanghai, China) in serum treated for 1 and 4 days. IL-6, IL-17A, IFN-γ, and TNF-α were detected according to the manufacturer’s protocol. Then samples were measured on a BD FACSCalibur Flow Cytometer and analyzed in FCAP Array Software Version 3.0 (BD Bioscience).

### Statistical Analysis

Each experiment was performed three independent times. Graphing and statistical analyses were performed in GraphPad Prism, version 7.0 (GraphPad Software Inc., La Jolla, CA, United States) with Student’s *t*-test. Cytokine data were compared with unpaired two-tailed Mann-Whitney (non-parametric) tests. Results represent the average of three independent experiments ± standard deviation (SD), and the level of statistical significance was set at *P < 0.05.

## Results

### Sodium New Houttuyfonate Has Antifungal Activity Against *A. fumigatus*

**FIGURE 1:**
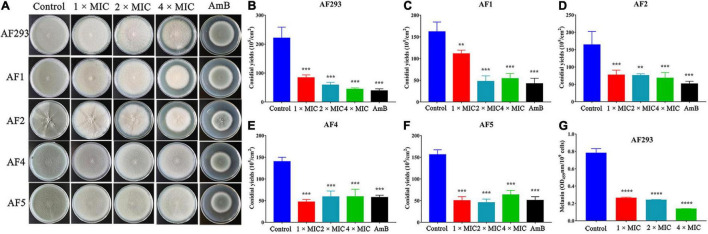
Sodium new houttuyfonate (SNH) inhibits the asexual cycle of *A. fumigatus*. **(A)** Images of various concentrations of SNH treatment on PDA plates. The *A. fumigatus* strains AF293, AF1, AF2, AF4, and AF5 were incubated at a concentration of 10^5^ cells/mL. Each dilution of 3 μL was deposited on PDA solid medium with 1 ×, 2 ×, or 4 × MIC SNH; AmB (8 μg/mL, positive control); or no drugs (negative control), then incubated at 37°C for 4 days. **(B–F)** Statistical analysis of *A. fumigatus* conidia yields under SNH treatment. **(G)** Measurement of *A. fumigatus* conidial melanin. Melanin was isolated from AF293 conidia cultured on PDA solid medium with 1 ×, 2 ×, or 4 × MIC SNH. Melanin was purified with 2% NaOH, and the absorbance at 459 nm was measured spectrophotometrically. Results represent the average of three independent experiments ± SD, and the level of statistical significance was set at *P < 0.05. **P < 0.01, ***P < 0.001, and ****P < 0.0001.

To evaluate the antifungal potential of SNH against *A. fumigatus*, we tested the MIC. The MIC of SNH against the AF293, AF1, and AF2 strains was 100 μg/mL, whereas that against AF4 and AF5 strains was 50 μg/mL. For AmB, the MIC for the AF293, AF1, and AF2 strains was 4 μg/mL, and was 0.5–1 μg/mL for AF4 and AF5. However, the FICI indicated that the combination ITR or VRC with SNH produced no synergistic effects against ITR- or VRC-resistant strains (data not shown). Furthermore, 1 ×, 2 ×, and 4 × MIC SNH against *A. fumigatus* was used to assess sporulation at 37 and 28°C. As shown in Figure 1 and Supplementary Figure 2, strains treated with SNH showed a significant decrease in conidial yield to 40–50% that in the untreated group (*P* < 0.05). Additionally, the *A. fumigatus* had a whitish color in the SNH treatment group. Further investigations confirmed that the conidia of SNH-treated strains accumulated less melanin than those of the control group (Figure 1G).

**FIGURE 2:**
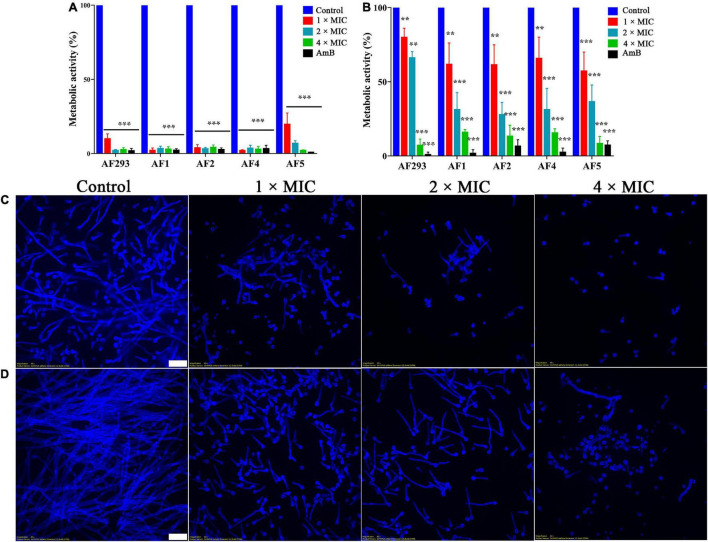
Effect of sodium new houttuyfonate (SNH) on biofilm. Readings of colorimetric XTT reduction assays at 492 nm are expressed as the percentage metabolic activity of the control. **(A)** Effect of SNH on the biofilm formation of the AF293, AF1, AF2, AF4, and AF5 strains. **(B)** Effect of SNH on preformed biofilms of the AF293, AF1, AF2, AF4, and AF5 strains. Results represent the average of three independent experiments ± SD, and the level of statistical significance was set at *P < 0.05, **P < 0.01, and ***P < 0.001. CLSM observation of the AF293 strain in **(C)** biofilm formation and **(D)** the preformed biofilm treated with 1 ×, 2 ×, and 4 × MIC SNH, respectively. Biofilms were stained with 1mL calcofluor white stain supplemented with 20 μL 10% KOH. Scale bar = 20 μm.

**FIGURE 3:**
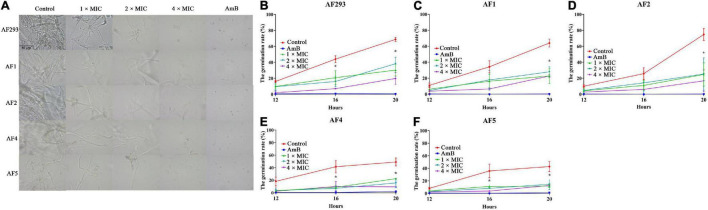
Effect of sodium new houttuyfonate (SNH) on conidial generation. **(A)** Generation of *A. fumigatus* treated with SNH. **(B–F)** *A. fumigatus* germination number counted after 1 ×, 2 ×, or 4 × MIC SNH treatment at 37°C for 12, 16, and 20 h. Testing of each group was performed in triplicate, and the level of statistical significance was set at *P < 0.05. The number of *A. fumigatus* germinations was counted under a microscope (400 ×).

The anti-biofilm activity of SNH was evaluated with XTT reduction analysis. The metabolic activity of biofilm was significantly decreased by treatment with even low concentrations of SNH (Figure 2A; *P* < 0.05). In the mature biofilms pretreated with 1 × MIC, XTT reduction assays demonstrated that the inhibition rate was approximately 20%. However, compared with the control treatment, 4 × MIC treatment resulted in an 80% lower inhibition rate (Figure 2B). Next, the fluorescent filamentous biomass of biofilms was examined by the CLSM. Compared with control group, as shown in Figures 2C,D, *A. fumigatus* mycelium length was shortened and the mature biofilm was thinner after SNH treatment. Hyphal growth, the basis of biofilm formation, was modest at 1 × and 2 × MIC; however, hyphal growth was absent at 4 × MIC (Figure 3). These results correlated with the results of the XXT reduction assays.

**FIGURE 4:**
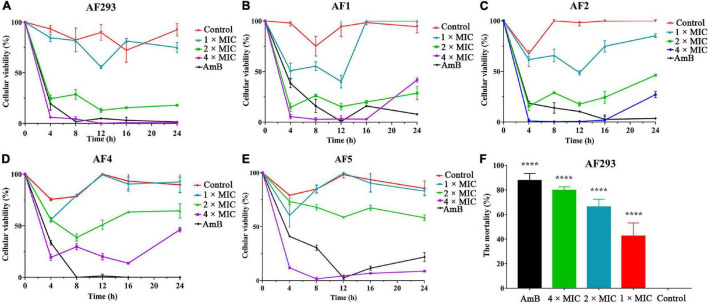
Sodium new houttuyfonate (SNH) has fungicidal activity against *A. fumigatus*. **(A–E)** Time-kill curves of 1 ×, 2 ×, 4 ×, and 8 × MIC SNH against *A. fumigatus* AF293, AF1, AF2, AF4, and AF5 strains. Readings of colorimetric XTT reduction assays at 492 nm are expressed as the percentage cellular viability of the control. **(F)** The mortality of AF293 was analyzed with PI staining after treatment with SNH at 1 ×, 2 ×, and 4 × MIC. AF293 treated with 8 μg/mL AmB and no drugs were used as the control groups. Results represent the average of three independent experiments ± SD, and the level of statistical significance was set at *P < 0.05, ****P < 0.0001.

Time-kill assays were used to investigate the kinetics of SNH against *A. fumigatus* (Figures 4A–E). Similar to amphotericin B (AmB), SNH exhibited fungistatic activity against all tested *A. fumigatus* strains at a concentration of 0.4 mg/mL. Further investigations were evaluated with PI staining. Statistical analysis indicated that the percentage of PI-stained cells rose to 42.37, 66.23, and 79.72% after treatment with SNH at concentrations of 0.1, 0.2, and 0.4 mg/mL, respectively (Figure 4F). In particular, the percentage of PI-stained cells treated with the 4 × MIC approached that in the AmB treatment group. These results revealed that SNH exhibited fungicidal activity against *A. fumigatus*.

### Sodium New Houttuyfonate Inhibits Ergosterol Synthesis Affecting Cell Membrane Integrity

**FIGURE 5:**
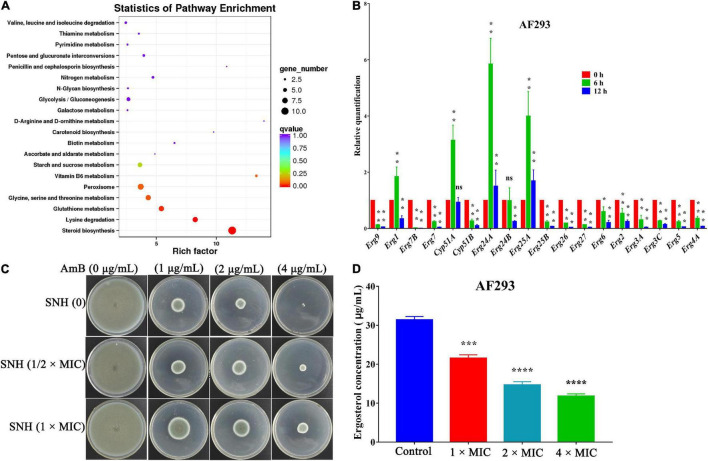
Antifungal mechanism of sodium new houttuyfonate (SNH). **(A)** Scatter plot of KEGG pathway enrichment statistics for the top 20 enriched pathways. The enrichment factor is the ratio of the number of genes that were differentially expressed to the total number of genes in a certain pathway. The color and size of the dots represent the range of the q-value and the number of DEGs mapped to the indicated pathways, respectively. **(B)** qRT-PCR analysis of gene expression levels. AF293 was incubated in the absence (control) or presence (2 × MIC) of SNH in RPMI-1640 medium at 37°C for 6 and 12 h. The histogram shows the relative expression of genes after SNH treatment with respect to the control. **(C)** *A. fumigatus* treated with SNH showed significantly diminished sensitivity to the AmB drug. AF293 conidia were treated with 0, 1/2 ×, and 1 × MIC SNH for 12 h at 37°C, then inoculated on PDA solid medium supplemented with AmB (0, 1, 2, and 4 μg/mL). **(D)** Quantification of ergosterol content. AF293 conidia were grown for 3 days in RPMI-1640 medium containing 0, 1 ×, 2 ×, and 4 × MIC SNH; ergosterol was extracted with 100% methanol; and spectral profiles were determined at 280 nm. Results represent the average of three independent experiments ± SD. The level of statistical significance was set at *P < 0.05. **P < 0.01, ***P < 0.001, and ****P < 0.0001; ns indicates no statistical difference.

After verifying SNH’s potent antifungal activity, we proceeded to investigate the antifungal mechanism of SNH. Transcriptomic changes in *A. fumigatus* treated with SNH were analyzed with Illumina sequencing, and 2.83 Gb clean data were obtained from each sample, with differential expression of 175 annotated genes. Raw sequence data have been deposited in the Beijing Institute of Genomics Genome Sequence Archive (accession number PRJCA007831). Genes with fold change ≥ 2 and false discovery rate (FDR) < 0.01 were considered significantly differentially expressed. The results showed that 66 genes were up-regulated and 109 genes were down-regulated (Supplementary Figure 3A). These differentially expressed genes (DEGs) were functionally grouped into gene ontology classes including 39 functional categories (Supplementary Figure 3B) and 16 clusters of orthologous groups (Supplementary Figure 3C). The DEGs were also assigned to 50 Kyoto Encyclopedia of Genes and Genomes (KEGG) pathways. The main KEGG enrichment pathway was the steroid biosynthesis pathway (Figure 5A).

To verify the transcriptomic results described above, we investigated genes associated with the ergosterol biosynthetic gene pathway through qRT-PCR analysis. In accordance with the transcriptomic data, the expression of 14 genes, including *Erg2* (C-8 sterol isomerase, putative), *Erg3* (Sterol desaturase), *Erg4* [C-24(28) sterol reductase], *Erg5* (Cytochrome P450 sterol C-22 desaturase, putative), *Erg6* (Sterol 24-c-methyltransferase, putative), *Erg7* (Oxidosqualene: lanosterol cyclase), *Erg26* (C-3 sterol dehydrogenase/C-4 decarboxylase), and *Erg27* (3-ketosteroid reductase), were down-regulated after SNH treatment. Furthermore, four other genes, *Erg1* (Squalene monooxygenase), *Erg24* (C-14 sterol reductase), *Erg25* (C-4 methyl sterol oxidase), and *Cyp51A* (14-alpha sterol demethylase), were up-regulated (Figure 5B). These data suggested that the antifungal activity of SNH against *A. fumigatus* involved interference with the steroid synthesis pathway. We further verified the antifungal mechanism on ergosterol biosynthesis by performing cell membrane stress assays. The strain treated with SNH showed significantly diminished sensitivity to AmB (Figure 5C). HPLC results (Figure 5D) were determined after treatment with SNH by 1 ×, 2 ×, and 4 × MIC, and the concentration of ergosterol decreased by 31.31, 53.25, and 62.42%, respectively. In summary, these data indicated that SNH inhibited the growth of *A. fumigatus* by inhibiting ergosterol synthesis.

### Sodium New Houttuyfonate Has Therapeutic Effects on Murine Invasive Aspergillosis

**FIGURE 6:**
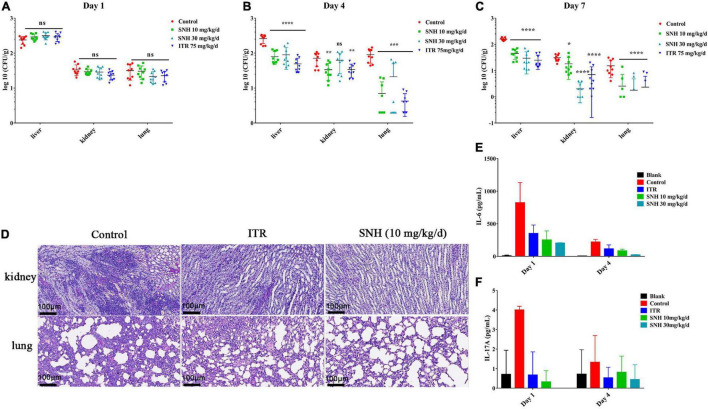
Therapeutic effect of sodium new houttuyfonate (SNH) on invasive aspergillosis (IA) in mice. **(A–C)** Fungal burden of the lung, kidney, and liver. The IA model mice were treated by gastric gavage with SNH at 10 mg/kg/d and 30 mg/kg/d, and the fungal burden was measured at 1–, 4–, and 7-days posttreatment. **(D)** Histological analysis of mice with IA. PAS-stained sections were prepared from the kidneys and lungs of mice 7 days after treatment with SNH at 10 mg/kg/d. Scale bar is 100 μm. **(E,F)** Determination of cytokines. Levels of IL-6 and IL-17A in various groups of mice serum on days 1 and 4 after treatment with SNH are shown. Each cytokine assay was performed three times, and the values represent average ± SD of three data points. Normal saline and ITR-treated IA mice were used in the control group, respectively. The statistical significance level was set at *P < 0.05. **P < 0.01, ***P < 0.001, and ****P < 0.0001; ns indicates no statistical significance compared with untreated mice with IA.

The IA murine model was used to assess the *in vivo* efficacy of antifungal activity. Compared with treatment with normal saline, treatment with SNH at doses of 10 mg/kg/day and 30 mg/kg/day significantly decreased the fungal burden in the liver, kidney, and lung (*P* < 0.05), and had effects equal to those of ITR from day 4 to day 7 (Figures 6A,C). Histopathology investigations using PAS-staining were consistent with the fungal burden results. Moreover, sections of PAS-stained kidney and lung were analyzed microscopically for abnormalities. The results revealed that the SNH or ITR treatment groups, compared with the treatment with normal saline, resulted in significantly fewer inflammatory cell infiltrates (Figure 6D), thus suggesting that repeated treatment with SNH was efficacious in IA mice.

To determine the effect of SNH on murine cellular immunity, we analyzed the serum of sacrificed mice 1 and 4 days after *A. fumigatus* challenge and drug treatment. Overall, the levels of IL-6 and IL-17A in mice with a treatment dose of SNH of 10 mg/kg/day or 30 mg/kg/day decreased (Figures 6E,F). On day 1, compared with the levels in untreated IA mice, the level of the pro-inflammatory cytokine IL-6 after treatment with SNH at a dose of 10 mg/kg/day was 3.26-fold lower, and that of IL-17A was 8-fold lower. After completion of 1 day of therapy in the SNH 30 mg/kg/day group, the level of IL-6 was decreased by 4.53-fold, and the level of IL-17A was undetectable. In addition, the levels of IFN-γ, and TNF-α were unchanged with respect to those in the sham control (Supplementary Figures 4B,C).

## Discussion

With fungal infections dramatically increasing worldwide, treatment efficacy is dependent on antifungal drugs. Unfortunately, the current antifungal agents are unsatisfactory. Drug repurposing is a promising technique to develop antifungal treatments, which has identified several non-antifungal agents with antifungal activity, such as antibacterial drugs, immunosuppressants, and statins (Kim et al., 2020; Zhang et al., 2021). SNH, initially used the treatment of respiratory infections, was also found to have anti-fungal activity against *C. albicans* (Shao et al., 2017; Wu et al., 2020). The current study demonstrated that SNH had fungistatic activity against *A. fumigatus* by inhibiting the synthesis of ergosterol. In addition, SNH exhibited potent *in vivo* antifungal activity against *A. fumigatus* infection.

Conidia are an important virulence factor of *A. fumigatus*. As many as 1,000 *A. fumigatus* conidia from the environment are inhaled into the alveoli daily (van de Veerdonk et al., 2017). The conidia can be cleared in healthy people. However, conidia can colonize the lungs of immunocompromised people and then germinate, thus causing invasive infections. Therefore, the suppression of conidia production and germination is essential to prevent *A. fumigatus* prophase infection. In our study, the conidiation and spore-to-hyphae morphological transition of *A. fumigatus* were significantly inhibited by treatment with even 1 × MIC of SNH in the spot dilution assays and in conidial germination assays (Figures 1 and 3). Moreover, lighter pigmentation levels and a whitish color of conidia were observed in the SNH treated group (Figure 1G). *A. fumigatus* conidia produce a bluish-green pigment in the cell wall to protect the fungus against the host immune defenses by shielding the fungal pathogen-associated molecular patterns or restricting the activation of reactive oxygen intermediates (Youngchim et al., 2004; Pihet et al., 2009; Schmaler-Ripcke et al., 2009; Chai et al., 2010). Notably, the albino conidia have been found to stimulate much stronger host defense mechanisms, such as increased levels of proinflammatory cytokines, such as TNF-α, IL-6, and IFN-γ (Chai et al., 2010). Furthermore, the ability to form biofilms is a unique aspect of fungal biology exploited by antifungal drugs. As much as 1,000-fold higher resistance has been estimated for pathogenic fungi in biofilms compared with the planktonic state (Rossoni et al., 2019; Zhang et al., 2021). The formation of hyphae is the basis for biofilm formation. In this study, SNH was found to effectively inhibit the biofilm formation of *A. fumigatus* and eradicate the formed biofilm (Figure 2), thus demonstrating that SNH had potent antifungal activity.

**FIGURE 7:**
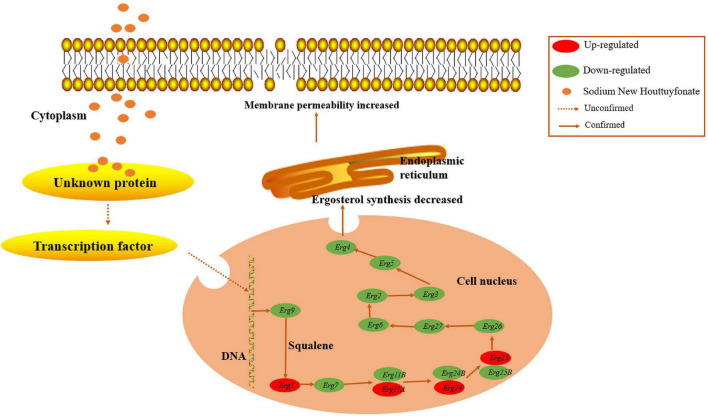
Model of the anti-*A. fumigatus* mechanism. Sodium new houttuyfonate (SNH) may activate an unknown protein or transcription factor, which translocates to the nucleus and inhibits ergosterol synthesis in the endoplasmic reticulum via suppressing the expression of related genes in *A. fumigatus*. The cell membrane permeability is elevated, thus contributing to cell growth inhibition or death. Dotted arrows indicate unknown pathways, and solid arrows indicate verified pathways.

Ergosterol, an essential element of the fungal cell membrane, forms direct linkages with the phospholipid membrane and plays an important role in membrane fluidity, cell cycle progression, cell morphology, and substance transportation (Banerjee et al., 2014; Rana et al., 2019). The biosynthesis of ergosterol is regulated by ergosterol biosynthetic enzymes. The ergosterol biosynthesis pathway is divided into three parts: the mevalonate pathway, ergosterol pathway, and toxic sterol pathway (Lan et al., 2021). The ergosterol pathway is generally chosen as a target for antifungal drug design. For instance, azole and allylamine decrease the production of ergosterol for fungistasis by inhibiting the *Erg11* and *Erg1* genes, respectively (Ryder, 1992; Quiles-Melero and García-Rodríguez, 2021). According to the transcriptomic results, the main KEGG enrichment pathway was steroid biosynthesis, thus suggesting that this pathway might be the target of SNH (Figure 5A). Herein, the expression of genes associated with ergosterol synthesis in the ergosterol pathway was analyzed. Most genes in this pathway were down-regulated, thus illustrating an inhibitory effect of SNH toward this pathway. Further results from cell membrane stress assays and HPLC confirmed our hypothesis. However, the expression of *Erg1*, *Erg11*, *Erg24*, and *Erg25* was up-regulated. We speculate that this up-regulation transitory compensatory reaction of *A. fumigatus* to the adverse external environment. In summary, we proposed a mechanistic model for the anti-*A. fumigatus* activity of SNH summarized in Figure 7 based on our current data. In this model, SNH may activate an unknown protein or transcription factor, which translocates to the nucleus and inhibits the ergosterol synthesis pathway. Cell membrane permeability is subsequently elevated, thus contributing to the inhibition of cell growth or inducing death. The underlying anti-*C. albicans* mechanism of SH and SNH have been reported to be closely involved with the synthesis and transport of β-1,3-glucan and the Ras1-cAMP-Efg1 pathway, respectively (Shao et al., 2017; Wu et al., 2020). However, in our transcriptomic data, the expression of these related genes showed no significant change (data not shown). How SNH affects the expression of ergosterol synthesis-related genes remains unknown and must be verified in further investigations.

A mouse model with IA was used to explore the therapeutic effects of SNH. The treatment of IA mice with SNH resulted in a lower fungal burden (Figures 6A–C) and less infiltration of inflammatory cells in tissues than were observed in the negative control (Figure 6D), thus suggesting that SNH was an effective therapy *in vivo*. Recently, the dysregulated production of Th cell cytokines has been confirmed to be associated with the pathogenesis of invasive aspergillosis (Cenci et al., 1999). Therefore, we investigated the levels of IL-6 and IL-17A produced by CD4^+^ Th cells (Figures 6E,F). In general, Th2 cytokines (e.g., IL-6) and Th17 cytokines (e.g., IL-17A) were associated with infection progression under conditions of compromised immunity, and the differentiation and immune function of Th17 cells was positively regulated by IL-6 (Egwuagu, 2009). In mice with IPA, excessive Th2-type reactivity has been found to be associated with poor prognosis in aspergillosis (Cenci et al., 2000). Moreover, Th1 cytokines (e.g., IFN-γ and TNF) are the key components of innate and adaptive immunity in anti-aspergillus defense (Singh et al., 2009; Park et al., 2009; Schmidt et al., 2011; Xu et al., 2018). Investigations have indicated that IFN-γ- and TNF-α- treated mice with IA show decreased mortality (Nagai et al., 1995). However, Th2 cells inhibited T cell activation and promoted the Th2 cell response by inhibiting proinflammatory cytokines and chemokines, thus preventing Th1-type reactivity in aspergillosis. However, in our investigation, all IA mice had high levels of IFN-γ and TNF-α, thereby suggesting a protective immune mechanism in the host. Further experiments are needed to elucidate the reason for the high levels of IFN-γ and TNF-α.

Together, the data from this study confirmed that SNH exhibited fungistatic activity and demonstrated that the effects of SNH on the ergosterol synthesis pathway inhibit *A. fumigatus* growth. However, SNH did not directly bind ergosterol and influence the cell membrane. Although further investigations are needed, the demonstration of the anti-*A. fumigatus* activity of IA *in vivo* supported that the SNH is a promising antifungal agent for the treatment of *A. fumigatus* infection.

## Data Availability Statement

The datasets presented in this study can be found in online repositories. The names of the repository/repositories and accession number(s) can be found in the article/Supplementary Material.

## Ethics Statement

The animal study was reviewed and approved by Mice experimental protocols were approved by the Southwest Medical University Institutional Animal Care and Use Committee (2020540).

## Author Contributions

ZS and YM conceived and designed the experiments. QZ and FL performed the experiments and drafted the manuscript. MZ analyzed the data and performed validation of relevant results. JZ and CX contributed to the revision of the manuscript. All authors read and approved the final version of the manuscript.

## Conflict of Interest

The authors declare that the research was conducted in the absence of any commercial or financial relationships that could be construed as a potential conflict of interest.

## Publisher’s Note

All claims expressed in this article are solely those of the authors and do not necessarily represent those of their affiliated organizations, or those of the publisher, the editors and the reviewers. Any product that may be evaluated in this article, or claim that may be made by its manufacturer, is not guaranteed or endorsed by the publisher.

## Supplementary Material

Supplementary Figure 1 Chemical structure of **(A)** sodium houttuyfonate, **(B)** sodium new houttuyfonate, **(C)** isavuconazole, **(D)** itraconazole, **(E)** posaconazole, and **(F)** voriconazole.

Supplementary Figure 2 Sodium new houttuyfonate (SNH) inhibits the asexual cycle of *A. fumigatus*. **(A)** Images of treatment with various concentrations of SNH on PDA plates. The *A. fumigatus* strains AF293, AF1, AF2, AF4, and AF5 were incubated at a concentration of 10^5^ cells/mL. Each 3 μL dilution was deposited on PDA solid medium with 1 ×, 2 ×, or 4 × MIC SNH; AmB (8 μg/mL, positive control); or no drugs (negative control) before incubation at 37°C for 4 days. **(B–F)** Statistical analysis of *A. fumigatus* conidia yields under SNH treatment. Results represent the average of three independent experiments ± SD, and the level of statistical significance was set at **P* < 0.05.

Supplementary Figure 3 Transcriptomic analyses. **(A)** Volcano plot of differentially expressed genes (DEGs). The green dots indicate downregulated genes (109) and the red dots indicate upregulated genes (66), with a total of 175 DEGs. **(B)** Gene ontology terms of DEGs. The distributions are summarized in three main categories: biological process (BP), molecular function (MF), and cellular component (CC). **(C)** Clusters of orthologous groups of the consensus sequence. The highest frequencies included carbohydrate transport and metabolism; secondary metabolites biosynthesis, transport, and catabolism; and amino acid transport and metabolism.

Supplementary Figure 4 Therapeutic effects of SNH on histologic analysis and determination of cytokines. **(A)** PAS-stained sections were prepared from the kidneys and lungs of mice at 4 days after treatment with SNH at 10 mg/kg/d. The black arrows indicated spores or hyphae. Scale bar is 100 μm. **(B,C)** Levels of IFN-γ and TNF-α in the serum in various groups of mice on days 1 and 4 after treatment with SNH are shown. The values represent the average ± SD of three data points. Normal saline and itraconazole (ITR)-treated IA mice were used in the control group.
