# Supplementary figures and images for: Antifungal Activity of Sodium New Houttuyfonate Against Aspergillus fumigatus in vitro and in vivo
Source: Front Microbiol. 2022 Apr 26;13:856272. doi: 10.3389/fmicb.2022.856272 (PMC9087332; doi:10.3389/fmicb.2022.856272)

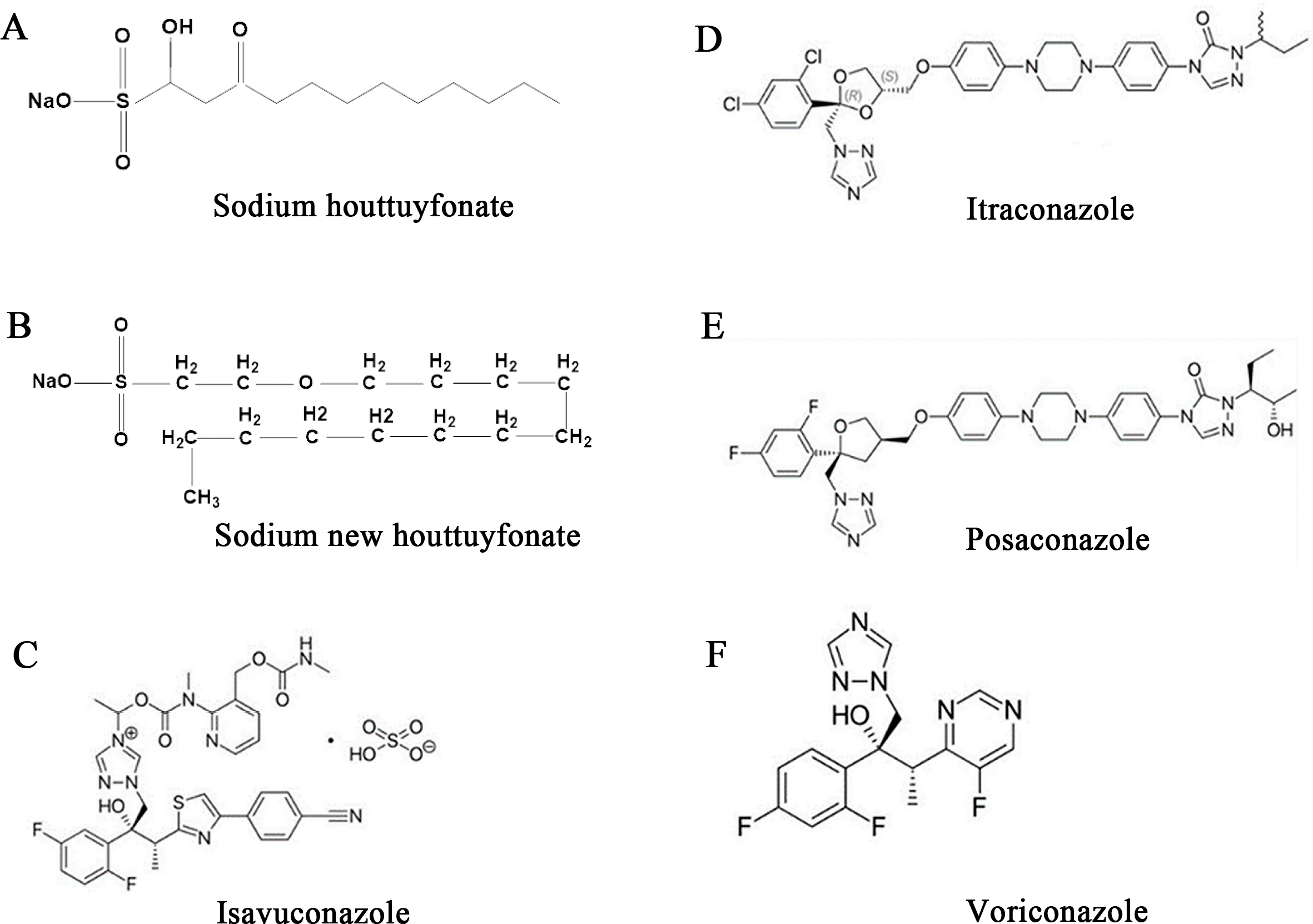

Supplement: Supplementary Figure 1 — Chemical structure of (A) sodium houttuyfonate, (B) sodium new houttuyfonate, (C) isavuconazole, (D) itraconazole, (E) posaconazole, and (F) voriconazole. [file Image_1.JPEG]

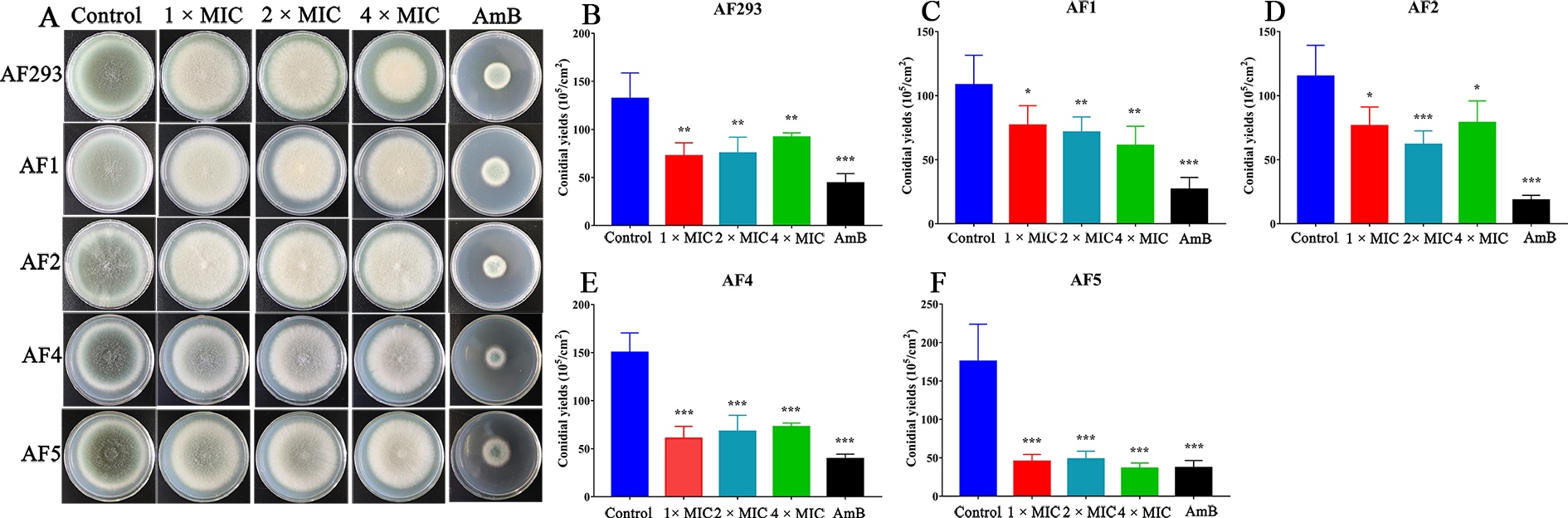

Supplement: Supplementary Figure 2 — Sodium new houttuyfonate (SNH) inhibits the asexual cycle of A. fumigatus. (A) Images of treatment with various concentrations of SNH on PDA plates. The A. fumigatus strains AF293, AF1, AF2, AF4, and AF5 were incubated at a concentration of 105 cells/mL. Each 3 μL dilution was deposited on PDA solid medium with 1 ×, 2 ×, or 4 × MIC SNH; AmB (8 μg/mL, positive control); or no drugs (negative control) before incubation at 37°C for 4 days. (B–F) Statistical analysis of A. fumigatus conidia yields under SNH treatment. Results represent the average of three independent experiments ± SD, and the level of statistical significance was set at *P < 0.05. [file Image_2.JPEG]

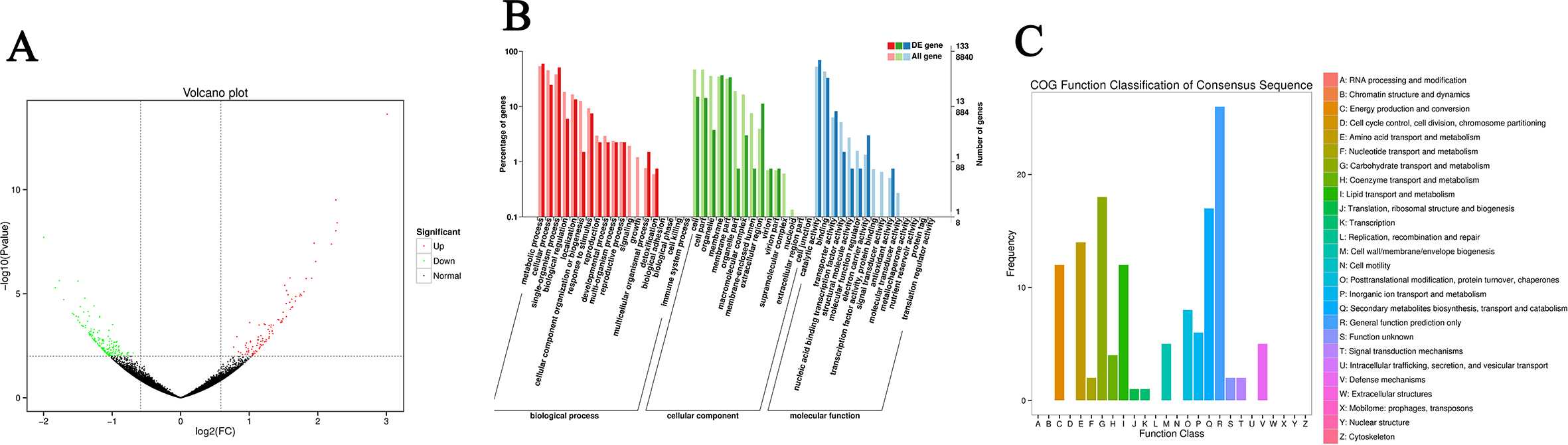

Supplement: Supplementary Figure 3 — Transcriptomic analyses. (A) Volcano plot of differentially expressed genes (DEGs). The green dots indicate downregulated genes (109) and the red dots indicate upregulated genes (66), with a total of 175 DEGs. (B) Gene ontology terms of DEGs. The distributions are summarized in three main categories: biological process (BP), molecular function (MF), and cellular component (CC). (C) Clusters of orthologous groups of the consensus sequence. The highest frequencies included carbohydrate transport and metabolism; secondary metabolites biosynthesis, transport, and catabolism; and amino acid transport and metabolism. [file Image_3.JPEG]

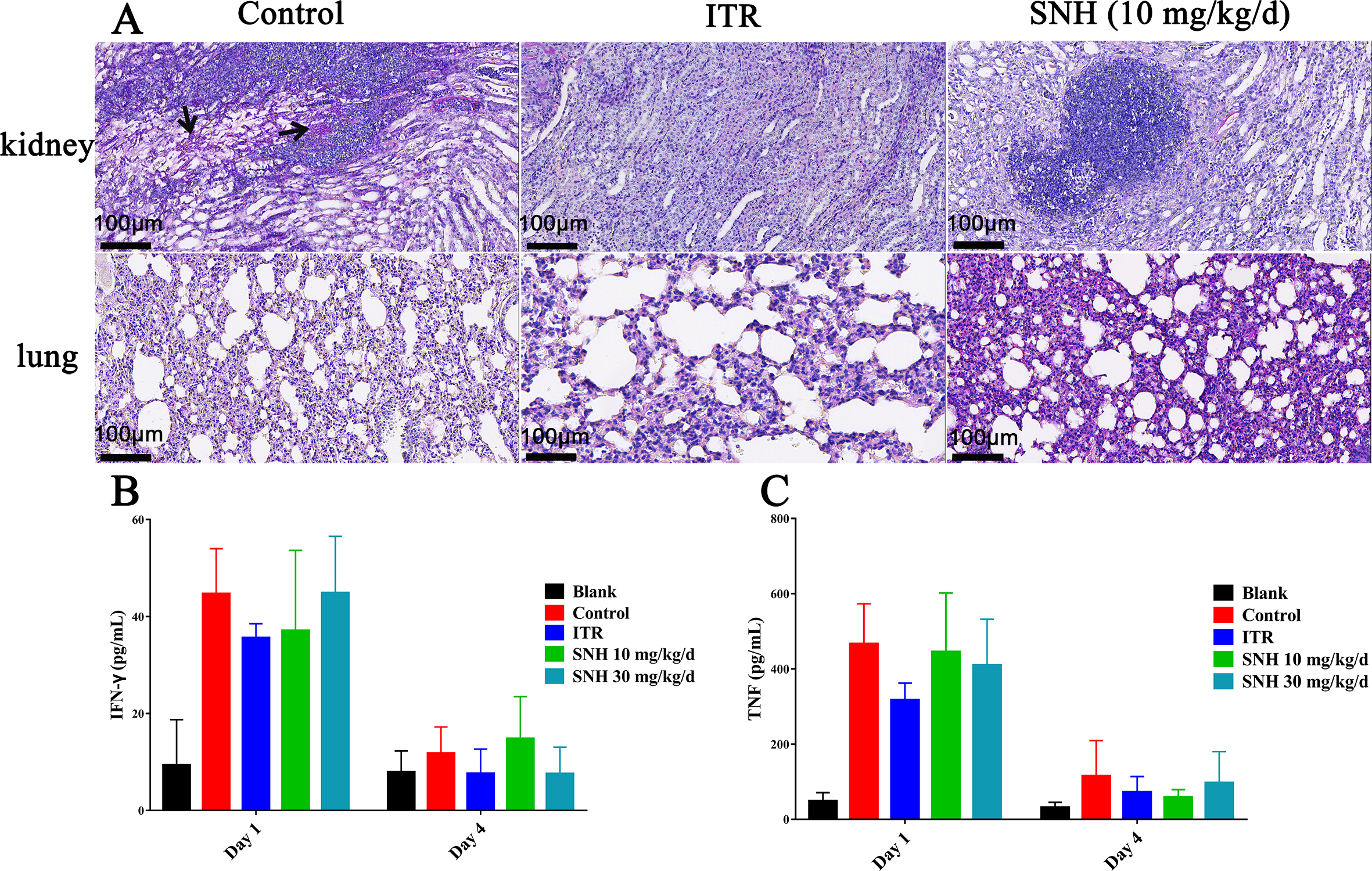

Supplement: Supplementary Figure 4 — Therapeutic effects of SNH on histologic analysis and determination of cytokines. (A) PAS-stained sections were prepared from the kidneys and lungs of mice at 4 days after treatment with SNH at 10 mg/kg/d. The black arrows indicated spores or hyphae. Scale bar is 100 μm. (B,C) Levels of IFN-γ and TNF-α in the serum in various groups of mice on days 1 and 4 after treatment with SNH are shown. The values represent the average ± SD of three data points. Normal saline and itraconazole (ITR)-treated IA mice were used in the control group. [file Image_4.JPEG]
