# Supplementary material for: Antifungal Activity of Sodium New Houttuyfonate Against Aspergillus fumigatus in vitro and in vivo
Source: Front Microbiol. 2022 Apr 26;13:856272. doi: 10.3389/fmicb.2022.856272 (PMC9087332; doi:10.3389/fmicb.2022.856272)
Supplement: Supplementary file 5 [file Table_1.DOCX]

**Supplementary Table 1**

Primers for qRT-PCR (ergosterol synthesis related genes).

| Accession number | Protein annotation | Gene | Oligo name | Sequence (5’ to 3’) |
| --- | --- | --- | --- | --- |
| Afu7g01220  Afu5g07780  Afu4g12040  Afu5g04080  Afu4g06890  Afu7g03740  Afu1g03150  Afu1g05720  Afu8g02440  Afu4g04820  Afu2g15030  Afu4g11500  Afu4g03630  Afu1g04720  Afu6g05140  Afu8g01070  Afu1g03950  Afu5g14350  Afua1g10910 | Farnesyl-diphosphate farnesyltransferase, putative  Squalene monooxygenase  Lanosterol synthase, putative  Oxidosqualene: lanosterol cyclase  14-alpha sterol demethylase  14-alpha sterol demethylase  C-14 sterol reductase  C-14 sterol reductase  C-4 methyl sterol oxidase  C-4 methyl sterol oxidase  C-3 sterol dehydrogenase/C-4 decarboxylase  3-ketosteroid reductase  Sterol 24-c-methyltransferase, putative  C-8 sterol isomerase, putative  Sterol delta 5,6-desaturase  Sterol desaturase, putative  Cytochrome P450 sterol C-22 desaturase, putative  C-24(28) sterol reductase  β-tubulin protein | *Erg9*  *Erg1*  *Erg7B*  *Erg7*  *Cyp51A*  *Cyp51B*  *Erg24*  *Erg24B*  *Erg25*  *Erg25B*  *Erg26*  *Erg27*  *Erg6*  *Erg2*  *Erg3A*  *Erg3C*  *Erg5*  *Erg4A*  *β-tubulin* | Af01220-QF  Af01220-QR  Af07780-QF  Af07780-QR  Af12040-QR  Af06890-QF  Af04080-QF  Af04080-QR  Af06890-QF  Af06890-QR  Af03740-QF  Af03740-QR  Af03150-QF  Af03150-QR  Af05720-QF  Af05720-QR  Af02440-QF  Af02440-QR  Af04820-QF  Af04820-QR  Af15030-QF  Af15030-QR  Af11500-QF  Af11500-QR  Af03630-QF  Af03630-QR  Af04720-QF  Af04720-QR  Af05140-QF  Af05140-QR  Af01070-QF  Af01070-QR  Af03950-QF  Af03950-QR  Af14350-QF  Af14350-QR  Af10910-QF  Af10910-QR | TCGAGACCATCTTCCCATCG  AATCACACCCATCAGAGCCA  TACCCTCCCTACCTCTTCCC  TATGGGAAGATGACGACGCA  CCAGCTTCCATACCAACTGC  TCCAAGCTGATGGAGCAGAA  TGCACATTGAGGCACATAGC  CAGAATTTCGCCCAGTGAGG  TCCAAGCTGATGGAGCAGAA  GTGAATCGCGCAGATAGTCC  GCGGGTCAACATTCTTCCTC  GAGGCAAGTCAGATCCGAGA  TTTAGCCCTGGGCACATACA  CTGGTGCCAATTCTCTCAGC  ATTAACCCGGCTTCTGGTGA  TTCCAGTCGGCACCATACTT  GACTTTGGTGGGCGCATTAT  ACAGCGGCTGAAGTAGATGA  GTCGTTTGTGATGCACGAGA  AAGGACAAACTTGGCGCAAT  ATAATCCTCGGTTGGCCGAT  GGCTTGACTTTGCGGAAGAT  TCGTGTAACCGTCTTGGTCA  TCTCTTCCGCAGTGAGATCC  TACGACAACGACAACCCTGA  AGTACCACGGAATGGCATCA  AGCTGGACCAGAAGATTCCC  TGTACATGGCACCCATAGCA  TGACCAATGCTGCCAATAGC  TCAGGTAGGCGTACTTGGAC  GATTCTGGTCCTCACGCAAC  CTCTTGGCGGACTTGATTCG  ACGAGAAGATTGCCAAGGGA  ACTCACGGAGCAAGTGAGAA  CATGTGACGTGTTCTTCGCT  ACCCGACGCTCATATTCCAT  TTCCCAACAACATCCAGACC  CGACGGAACATAGCAGTGAA |
